# Cancer susceptibility genes: update and systematic perspectives

**DOI:** 10.1016/j.xinn.2022.100277

**Published:** 2022-06-28

**Authors:** Xiaoshun Shi, Sylvia Young, Kaican Cai, Jialiang Yang, Grant Morahan

**Affiliations:** 1 Harry Perkins Institute of Medical Research, QEII Medical Centre and Centre for Medical Research, The University of Western Australia, Nedlands, Perth, WA 6009, Australia; 2 Department of Thoracic Surgery, Nanfang Hospital, Southern Medical University, Guangzhou 510515, China; 3 Qingdao Geneis Institute of Big Data Mining and Precision Medicine, Qingdao 266000, China

A cancer susceptibility gene (CSG) is a gene whose variants can increase the cancer risk of those bearing such variants.^1^ By defining an individual’s CSG profile, it may be possible to assess their cancer risk and may provide insights for future prevention and treatment of cancer.

The methods used for discovery of CSGs have evolved from single candidate gene analysis to population-based next-generation sequencing. The syndrome of increased familial susceptibility to neoplastic diseases was described in 1969 by Li and Fraumeni, and by gene linkage within affected families followed by association analysis of candidate genes in the linkage region, the causative gene, p53, was identified. Such studies are expensive, difficult in terms of patient recruitment, and time consuming, so few CSGs had been identified by 2000.

Population-based CSG candidate discovery was facilitated by the breakthrough of widespread genotyping, allowing genome-wide association studies. Further advances in whole-exome and whole-genome sequencing allowed identification of genetic variants by comparing their frequency in population-based analyses of patients with cancer and control subjects. However, these methods are costly and limited in practical use. Compared with whole-genome and whole-exome sequencing, panel sequencing by reducing genome-wide genes to cancer-associated or customized target genes is less costly and more widespread in clinical genetic testing. Although these studies permit significantly expanded sample sizes, they are limited to studying known genes.

Most CSGs discovered by the above methods have not been proven to cause cancer and have not yet been applied clinically, but a comprehensive summary of the current CSGs is helpful to acknowledge the progress of the field, to provide a reference gene list for genetic consulting, and for mining potential key cancer-prevention targets. Bioinformatic analysis has an emerging role in CSG discovery. In this overview, efforts have been made to summarize the CSG list associated with the risk of cancer (https://csgs.sequenxe.com/), including those from literature review,^1^ large-scale analysis of predisposition variants in cancer,^2^ the GWAS Catalog database,^3^ and manual database retrieval. The resulting list of CSGs increased six-fold from the 114 CSGs^1^ reported in 2014 and accounts for up to 3.5% of human protein-coding genes.^4^

While CSGs are spread across every human chromosome,^1^ in the updated list we found a higher density in particular chromosomal regions. The ratio of CSGs to genes in a chromosome ranges from 0.17% on chromosome Y to 1.45% on chromosome 6. Chromosome 1 harbors the most CSGs since it is the largest human chromosome. No CSGs on chromosome Y have been identified since 2014.

**Figure 1:**
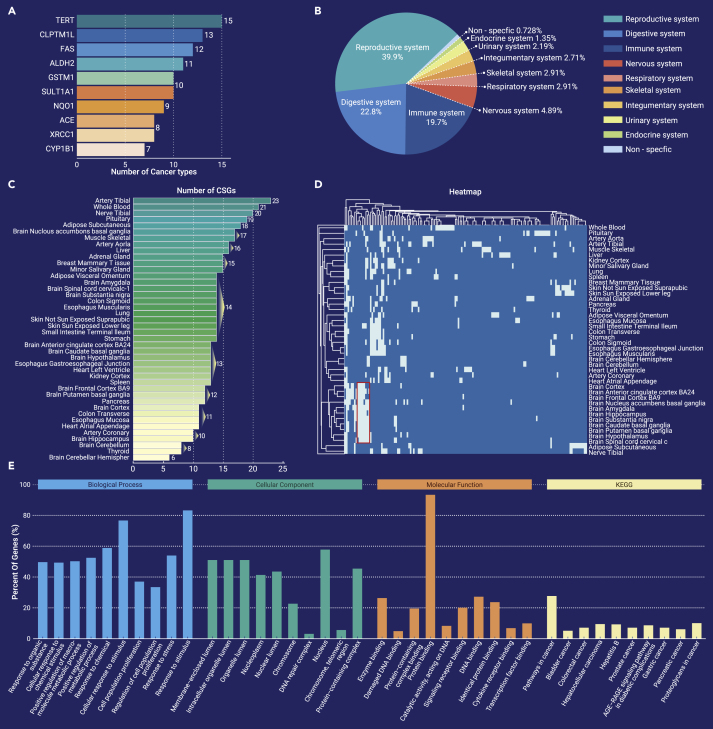
Systematic perspectives of current CSGs (**A**) Examples of CSGs associated with multiple cancer types. (**B**) Distribution of CSGs in tissue systems. (**C**) Number of sex-differentially expressed CSGs by tissue types. (**D**) Sex-specific CSGs with differential expression in human tissues. CSGs that were not differentially expressed by sex in any of the tissues were not included in the analysis. Example of sex-differential expression of CSGs in specific tissue such as brain was highlighted in red box. (**E**) Assessment of pathways affected by CSGs. The update CSGs were submitted to g:Profiler for pathway analysis, and the top 10 pathways were visualized.

A summary of which CSGs are associated with most cancer types may assist in identifying cancer-prevention targets. We examined the number of cancer types with which each CSG is associated. Currently, many CSGs are associated with multiple cancer types. (Figure 1A). Further analysis of the biological function of the highlighted genes is needed to understand the mechanisms of oncogenesis by which these genes contribute to the risk of multiple cancers.

It is not known which systems are more affected by inherited genetic mutations. We first classified cancer types based on the human anatomical system; for example, cancers in the reproductive system include breast cancer and prostate cancer, and CSGs with known associations in cancer types such as *BRCA2* and breast cancer, *APC* and colon cancer, and *RB1* and retinoblastoma were included. Then, the number of CSGs predisposing an individual to cancer in this system was summed. By considering the organ systems affected by CSGs, the most-affected organs were in the reproductive system (notwithstanding the increased analyses performed on breast cancer), followed by the digestive and immune systems. (Figure 1B). Why do these systems have a more heritable risk of cancer? Although CSGs in DNA repair, environmental factors, and immune function against cancer could be involved, understanding cancer susceptibility in each anatomical system could offer a new angle of how inherited genetic mutations act in a specific human system and provide hints for future CSG studies.

To our knowledge, the sex-specific CSG expression profile across human tissues has not been reported. By mapping CSGs to the recent sex-differential transcriptome,^5^ we found that 77% of CSGs were not sex-differentially expressed in reported human tissue. The average number of CSGs with sex-differential expression across human tissues was 14, ranging from six in the brain cerebellar hemisphere to 23 in the tibial artery (Figure 1C). We also observed that while some CSGs were sex-differentially expressed across most human tissue (*PAX8* and *CDKN2A*), some were sex-differentially expressed only within specific tissues, such as *GSTP1* and *CASP8* in brain (Figure 1D, red box). These findings suggest that some CSGs could predispose patients to cancer in a sex-specific manner and could further be limited to specific cancer types.

Studies of common pathways in which CSGs are involved may improve our mechanistic insights into cancer origin. Here, we provide a different aspect of common pathways affected by CSGs with a background of all genes compared with previous findings. In a Kyoto Encyclopedia of Genes and Genomes analysis, the evidence of CSGs affecting “pathways in cancer” was solid (Figure 1E). Furthermore, the top Gene Ontology terms that CSGs *TP53* and *BRCA2* were enriched in were “response to an organic substance,” “enzyme binding,” and “membrane-enclosed lumen.” This suggests that the malfunction within cells of the molecular binding ability of external factors could be a general susceptibility mechanism. By mapping the CSG list to any cancer-causing signaling pathways, such as the MAPK signaling pathway, we could identify which genetic node in tumorigenic pathways could mediate cancer susceptibility.

Studies of CSGs continue, and the number of CSGs is expanding. With advanced computational tools applied in medical research such as machine-learning and deep neural networks, more CSGs will be identified. Our knowledge of how individual CSGs contribute to susceptibility in people bearing risk alleles will improve our understanding of inherited cancer susceptibility and eventually lead to personalized cancer prevention and treatments.
